# 

**Published:** 1846-10-01

**Authors:** 


### CONTENTS

OF THE

### MEDICO-CHIRURGICAL REVIEW,

### OCTOBER 1, 1846.

### REVIEWS.

I.

Rapport a l'Academie Royale de Medécine sur la Peste et les Quarantaines, fait, au nom d'une Commission, par M. le Dr. Prus .. .. .. .. .. .. .. 285
1. Various sources of Information .. .. .. .. .. .. .. .. 288
2. The Countries where the Plague has been indigenous .. .. .. .. 290
3. The Predisposing and Exciting Causes of the Plague .. .. .. .. 293
4. The Plague endemic in Lower Egypt .. .. .. .. .. .. .. .. 296
5. The Epidemic Characters of the Plague.. .. .. .. .. .. .. .. 298
6. Difference between Sporadic and Epidemic Plague .. .. .. .. 305
7. Mode of Diffusion of the Plague .. .. .. .. .. .. .. .. .. 306
8. Is the Plague transmissible by Inoculation? .. .. .. .. .. .. .. 308
9. Is it transmissible by contact with the Sick? .. .. .. .. .. .. 310
10. Is it transmissible by Clothes, Merchandize, &c. .. .. .. .. .. 314
11. Is it transmissible through the medium of the Air? .. .. .. .. 316
12. Is it transmissible beyond Epidemic Foci? .. .. .. .. .. .. .. 321
13. By what circumstances is the Transmissibility influenced?.. .. .. 326
14. Little risk of imported Plague becoming Epidemic .. .. .. .. 327
15. Term of the Incubation of the Plague .. .. .. .. .. .. .. .. 329
16. Changes proposed in Quarantine Regulations .. .. .. .. .. 331

II.

Nouvelles Recherches sur les Bruits des Artères et Application de ces Recherches a l'Etude de plusieurs Maladies. Par le Dr. J. H. S. Beau.. .. .. .. .. 333
1. History of Arterial Murmurs.. .. .. .. .. .. .. .. .. .. .. 333
2. The various Diseases which are characterised by the existence of Arterial Murmurs .. .. .. .. .. .. .. .. .. .. .. .. 338
  A. Insufficiency of the Valves of the Aorta .. .. .. .. .. .. 339
  B. Anæmia following Loss of Blood .. .. .. .. .. .. .. .. 339
  C. Chlorosis .. .. .. .. .. .. .. .. .. .. .. .. .. .. 342
  D. The Cachexiæ .. .. .. .. .. .. .. .. .. .. .. .. 343
  E. The Menstrual Epoch—Amenorrhœa—Pregnancy .. .. .. 346
  F. Fevers .. .. .. .. .. .. .. .. .. .. .. .. .. .. 347
  G. Scorbutus and Purpura .. .. .. .. .. .. .. .. .. .. 348
  H. Dropsies .. .. .. .. .. .. .. .. .. .. .. .. .. 348
  I. Hypochondriasis .. .. .. .. .. .. .. .. .. .. .. .. 349
3. General Conclusions .. .. .. .. .. .. .. .. .. .. .. 350
  A. Therapeutical Indications.. .. .. .. .. .. .. .. .. .. 353

III.

I. Clinical Illustrations of the Diseases of India, &c. By WILLIAM GEDDES, M.D. &c... .. .. .. .. .. .. .. .. .. .. .. .. .. .. } 355

II. Remarks on the Dysentery and Hepatitis of India. By C. A. PARKES, M.D. }

1. Intermittent and Remittent Fevers.. .. .. .. .. .. .. .. .. 358

2. Continued and Ephemeral Fevers .. .. .. .. .. .. .. .. .. 367

3. Diseases of the Head—Phrenitis ; Delirium Tremens .. .. .. .. 368

4. Abdominal Inflammation—Splenitis ; Colitis, &c .. .. .. .. .. . 369

5. Hepatic Inflammation and Abscess.. .. .. .. .. .. .. .. .. 370

6. Dysentery .. .. .. .. .. .. .. .. .. .. .. .. .. .. 376

7. Rheumatism .. .. .. .. .. .. .. .. .. .. .. .. .. 382

IV.

A Practical Treatise on the Diseases of Children. By JAS. MILMAN COLEY, M.D. 386
1. Effects of Dentition .. .. .. .. .. .. .. .. .. .. .. .. 388
2. Erysipelas of Infants .. .. .. .. .. .. .. .. .. .. .. 389
3. Furunculus or Boil .. .. .. .. .. .. .. .. .. .. .. .. 390
4. Porrigo Scutulata, or Ringworm of the Scalp .. .. .. .. .. .. 390
5. Dysentery in Children .. .. .. .. .. .. .. .. .. .. .. 391
6. Treatment of Pertussis .. . .. .. .. .. .. .. .. .. .. 391
7. Plugging the Nares in Epistaxis .. .. .. .. .. .. .. .. 392

V.

Observations on the principal Hospitals for the Insane in Great Britain, France, and Germany. By J. RAY, M.D . .. .. .. .. .. .. .. .. .. .. 392
1. The Visitation and Direction of Asylums .. .. .. .. .. .. .. 394
2. Officers .. .. .. .. .. .. .. .. .. .. .. .. .. .. 395
3. Site and Construction of European Asylums.. .. .. .. .. .. .. 397
4. Sleeping-rooms and Dormitories .. .. .. .. .. .. .. .. .. 397
5. Day-rooms and Padded-rooms.. .. .. .. .. .. .. .. .. .. 399
6. Warming and Ventilation .. .. .. .. .. .. .. .. .. .. 399
7. Attendants .. .. .. .. .. .. .. .. .. .. .. .. .. 399
8. Restraint and Non-restraint .. .. .. .. .. .. .. .. .. .. 401
9. Occupations and Amusements .. .. .. .. . .. .. .. .. 401
10. Schools .. .. .. .. .. .. .. .. .. .. .. .. .. .. 402
11. Medication.. .. .. .. .. .. .. .. .. .. .. .. .. .. 403
12. Religious Exercises .. .. .. .. .. .. .. .. .. .. .. 403

VI.

The Surgical, Mechanical, and Medical Treatment of the Teeth, &c. By JAMES ROBINSON .. .. .. .. .. .. .. .. .. .. .. .. .. .. .. .. 404

VII.

Commentary on the Hindu System of Medicine. By T. WISE, M.D. &c. .. . 411
1. Duties and Rewards of Medical Men .. .. .. .. .. .. .. .. 413
2. Hygienic Regimen, Baths, Diet, &c. .. .. .. .. .. .. .. .. 415
3. Original *habitat* of Small-pox.. .. .. .. .. .. .. .. .. .. 416
4. Leprosy ; Demoniac Possessions, &c. .. .. .. .. .. .. .. .. 417

VIII.

I. A Manual of Operative Surgery. By J. MALGAIGNE .. .. .. .. .. .. } 419
II. Practical Surgery. By ROBERT LISTON.. .. .. .. .. .. .. .. }
1. Bleeding from the Foot .. .. .. .. .. .. .. .. . .. .. 423
2. Opening Abscesses by Incision .. .. .. .. .. .. .. .. .. 424
3. Lithotrity and Lithotomy .. .. .. .. .. .. .. .. .. .. 425
4. Inguinal Hernia.. .. .. .. .. .. .. .. .. .. .. .. .. 428
5. Operation without opening the Sac .. .. .. .. .. .. .. .. 430
6. Femoral Hernia .. .. .. .. .. .. .. .. .. .. .. .. 430
7. Dilatability of the Vagina .. .. .. .. .. .. .. .. .. .. 431

IX.

Deontologie Medicale, ou des Devoirs et des Droits des Medecins, dans l'Etat actuel de la Civilisation. Par le Docteur MAX. SIMON .. .. .. .. .. .. .. 431

1. The Moral Duties of our Profession .. .. .. .. .. .. .. .. .. .. 433
2. The Colleges of Physicians and Surgeons censured .. .. .. .. .. 435
3. The Materialism of Medical Studies.. .. .. .. .. .. .. .. .. .. 437
4. Experiments on Living Animals condemned .. .. .. .. .. .. 443

X.

I. The Brain and its Physiology. By DANIEL NOBLE, M.R.C.S.E. .. ..
II. Examen de la Phrénologie. Par P. FLOURENS .. .. .. .. .. .. ..
III. Quelques Considérations en Réponse a l'Examen de M. le Professeur P. Flourens. Par M. S. de WOLKOFF .. .. .. .. .. .. .. .. .. } 447
1 Value of Gall's Cerebral Anatomy.. .. .. .. .. .. .. .. .. .. 449
2. Cranioscopy of Carus .. .. .. .. .. .. .. .. .. .. .. .. .. 453
3. Deductions from Cerebral Pathology .. .. .. .. .. .. .. .. .. 455
4. Fallacies of Gall and Spurzheim .. .. .. .. .. .. .. .. .. .. 457
5. The Function of the Cerebellum .. .. .. .. .. .. .. .. .. .. 459
6. Connexion between Phrenology and Insanity .. .. .. .. .. .. 461
7. Estimate of Phrenology .. .. .. .. .. .. .. .. .. .. .. .. .. 463

XI.

I. Delle Alterazioni Pathologiche delle Arterie per la Legatura et la Torsione. Esperienze et Osservazioni di LUIGI PORTA, Professore di Clinica Chirurgica nell' I. R. Università di Pavia .. .. .. .. .. .. .. .. ..
II. On Wounds of the Arteries of the Human Body, with the Treatment and Operations required for their Cure. By G. J. GUTHRIE, F.R.S. .. .. } 464
1. Anatomy of the Arteries.. .. .. .. .. .. .. .. .. .. .. .. .. 465
2. On the Application of the Ligature .. .. .. .. .. .. .. .. .. 466
3. On the Torsion of Arteries .. .. .. .. .. .. .. .. .. .. .. .. 469
4. On the Collateral Circulation established after the Obliteration of the Arterial Trunks .. .. .. .. .. .. .. .. .. .. .. .. .. .. 470
5. Treatment of Wounds of Arteries.. .. .. .. .. .. .. .. .. .. 475

XII.

The Economy of the Animal Kingdom considered Anatomically, Physically, and Philosophically. By EMANUEL SWEDENBORG .. .. .. .. .. .. .. .. 478

XIII.

I. Animal Chemistry, with reference to the Physiology and Pathology of Man. By Dr. FRANZ. SIMON .. .. .. .. .. .. .. .. .. .. .. ..
II. Dr. DAY's Reports on the Progress of Animal Chemistry .. .. .. ..
III. Grundlinien der Physiologischen und Pathologischen Chemie. Fur Aerzte und Studirende. Von Dr. HERMANN HOFFMAN .. .. .. .. .. ..
IV. A Practical Manual, containing a Description of the General, Chemical, and Microscopic Characters of the Blood and Secretions of the Human Body, as well as their Components, &c. By JOHN WILLIAM GRIFFITH, M.D. F.L.S &c... .. .. .. .. .. .. .. .. .. .. .. .. .. .. .. .. .. } 481
1. Changes of the Blood in Inflammation .. .. .. .. .. .. .. .. 482
2. Colouring Matters of the Blood, Bile and Urine .. .. .. .. .. 485
3. Liebig's Views respecting the Bile disputed .. .. .. .. .. .. 487
4. Peculiar Forms of Blood-Corpuscles .. .. .. .. .. .. .. .. 489
5. General Chemical Relations of the Blood .. .. .. .. .. .. .. 490
6. Healthy and Morbid Conditions of the Blood .. .. .. .. .. .. 493
7. Chemistry of the Bile, Gastric Juice, and Miik .. .. .. .. .. 494
8. Pathological Relations of the Urine .. .. .. .. .. .. .. .. 497
9. Chemistry of various Morbid Products.. .. .. .. .. .. .. .. 499

XIV.

I. The Why and the Wherefore, or the Philosophy of Life, Health, and Disease. By CHARLES SEARLE, M.D., M.R.C.S.E. .. .. .. .. .. .. .. ..
II. Liebig's Physiology applied in the Treatment of Functional Derangement and Organic Disease. By JOHN LEESON .. .. .. .. .. .. .. .. ..
III. Homœopathy, Allopathy, and "Young Physic." By JOHN FORBES, M.D., F.R.S. .. .. .. .. .. .. .. .. .. .. .. .. .. .. .. .. } 501

XV.

I. On the Nerves of the Uterus. By Thomas Snow Beck, Esq., Surgeon .. } 514
II. Supplement to a Paper "On the Nervous Ganglia of the Uterus." By Robert Lee, M.D., &c. .. .. .. .. .. .. .. .. .. .. .. .. .. }
1. Relations of Sympathetic Nerve .. .. .. .. .. .. .. .. .. .. 517
2. Controversy respecting the Size of the Uterine Nerves .. .. .. 518

XVI.

The Sanative Influence of Climate. By Sir James Clark, Bart., M.D., F.R.S. .. 520

XVII.

The Microscopic Anatomy of the Human Body, in Health and Disease. By Arthur Hill Hassall .. .. .. .. .. .. .. .. .. .. .. .. .. .. .. 523
Action of the Red Particles in Respiration .. .. .. .. .. .. .. 524

XVIII.

Correspondence respecting the Quarantine Laws. Presented to Parliament .. 525
1. Evidence of Sir William Pym.. .. .. .. .. .. .. .. .. .. .. .. 526
2. Official Reports from the Lazarettos of Malta and Marseilles .. .. 528

### PERISCOPE.

### Selections from the Foreign Periodicals.

1. New Researches on the Composition of the Blood in Health and Disease. By MM. A. Becquerel and A. Rodier .. .. .. .. .. .. .. .. .. .. .. .. .. 533
2. The Cæsarian Operation considered in a Theological Point of View .. .. 537
3. On the Treatment of Photophobia. By Dr. Duval .. .. .. .. .. .. .. .. 538
4. Cause of Death in the Aged .. .. .. .. .. .. .. .. .. .. .. .. .. .. .. 540
5. Velpeau on Fracture of the Olecranon and Patella .. .. .. .. .. .. .. 540
6. Seat of Abscess after Fractures, &c. .. .. .. .. .. .. .. .. .. .. .. .. 541
7. Paralysis of the Third Pair of Nerves in Facial Neuralgia .. .. .. .. .. .. 543
8. Amaurosis from Injury of the Frontal Nerve .. .. .. .. .. .. .. .. .. 544
9. Dr. Dubini on Electrical or Acute Chorea .. .. .. .. .. .. .. .. .. .. 545
10. Treatment of Fracture of the Clavicle.. .. .. .. .. .. .. .. .. .. .. .. 548
11. Nitrate of Silver in Traumatic Erysipelas, Arthritis, &c. .. .. .. .. .. .. 548
12. M. Velpeau on Flexions and Engorgements of the Uterus.. .. .. .. .. .. 548
13. On Paralysis of the Insane. By M. Baillarger .. .. .. .. .. .. .. .. .. 549
14. Cancer .. .. .. .. .. .. .. .. .. .. .. .. .. .. .. .. .. .. .. .. .. 552
15. Ergotine as a Hæmostatic .. .. .. .. .. .. .. .. .. .. .. .. .. .. .. 552
16. M. Ricord on the Treatment of Gonorrhœa .. .. .. .. .. .. .. .. .. 553
17. M. Gintrac on Diagnosis of Chlorosis .. .. .. .. .. .. .. .. .. .. .. .. 556

### Bibliographical Notices, &c.

The Harveian Oration, delivered before the Royal College of Physicians, London, June 27th, 1846. By John Elliotson, M.D., &c. .. .. .. .. .. .. .. .. 557
Mesmerism in India, and its Practical Application in Surgery and Medicine. By James Esdaile, M.D. .. .. .. .. .. .. .. .. .. .. .. .. .. .. .. .. 558
Fever Physiologically considered. By David M'Connell Reed, Esq. .. .. }
Annual Report of the Cork Fever Hospital for the Year 1845 .. .. .. .. .. } 559
Medical Report of the House of Recovery and Fever Hospital, Cork Street, Dublin. By George Kennedy, M.D., &c. .. .. .. .. .. .. .. .. .. }
On the Antidotal Treatment of the Epidemic Cholera. By John Parkin, M.D. .. 559
Observations on the Edinburgh Pharmacopœia, and on the Dispensatories of Dr. Christison and Dr. A. T. Thomson. By Richard Phillips, F.R.S., &c. .. 560
A Medical Topography of Tunbridge Wells. By Robert Hutchinson Powel, M.B. M.D. .. .. .. .. .. .. .. .. .. .. .. .. .. .. .. .. .. .. .. 561
Quain on the Arteries .. .. .. .. .. .. .. .. .. .. .. .. .. .. .. .. .. .. 561

